# Extraction of features from sleep EEG for Bayesian assessment of brain development

**DOI:** 10.1371/journal.pone.0174027

**Published:** 2017-03-21

**Authors:** Vitaly Schetinin, Livija Jakaite

**Affiliations:** School of Computer Science, University of Bedfordshire, Park Square, Luton, LU1 3JU, United Kingdom; Centro de Neurociencias de Cuba, CUBA

## Abstract

Brain development can be evaluated by experts analysing age-related patterns in sleep electroencephalograms (EEG). Natural variations in the patterns, noise, and artefacts affect the evaluation accuracy as well as experts’ agreement. The knowledge of predictive posterior distribution allows experts to estimate confidence intervals within which decisions are distributed. Bayesian approach to probabilistic inference has provided accurate estimates of intervals of interest. In this paper we propose a new feature extraction technique for Bayesian assessment and estimation of predictive distribution in a case of newborn brain development assessment. The new EEG features are verified within the Bayesian framework on a large EEG data set including 1,100 recordings made from newborns in 10 age groups. The proposed features are highly correlated with brain maturation and their use increases the assessment accuracy.

## Introduction

Expert evaluation of brain development is mainly carried out by analysing age-related patterns in sleep electroencephalograms (EEG), represented by different characteristics such as waves, amplitude distributions, and variations over sleep stages, that reflect the non-stationary nature of EEG, see e.g. [1, 2]. For quantitative analysis, EEG data are split into segments within which changes are not significant and EEG can be considered as quasi-stationary signals. The duration of such intervals is typically between 2 and 20 sec, [1, 3].

Despite the wide variability of sleep EEG, there have been identified patterns for newborns at different post-conception weeks (ages), that allow experts to evaluate EEG maturity with the accuracy of ±1 week, see e.g. [3, 4]. When brain development is normal, the EEG evaluation typically matches the newborn’s age, whilst in pathological cases the EEG evaluation mismatches the age. The results of evaluations however can be heavily affected by EEG artefacts, noise as well as by the variability of the age-related patterns.

One of important patterns for EEG evaluation is the *discontinuity* that is represented by amplitude and frequency changes. An EEG pattern is defined discontinuous if an interval with a voltage above the normal value is interchanged with a period of a low voltage. The discontinuity in EEG of newborns between 28 and 30 weeks contains high-amplitude bursts visible as waves of mixed frequencies. These bursts are interchanged by long low-voltage periods. After 30 weeks, the variability of amplitudes decreases and periods of an uninterrupted EEG activity become longer, and the discontinuity is progressively decreased, see e.g. [3, 5, 6]

In practice of EEG evaluation, reference guidances have not been established as the discontinuity is difficult to measure quantitatively, see e.g [7]. Automated estimation of the discontinuity has been attempted with a threshold segmentation technique proposed in [8]. However, a threshold required for such segmentation is heavily dependent on EEG characteristics that widely vary between patients as well as during sleep hours.

Adaptive segmentation has been proposed in order to find pseudo-stationary intervals in EEG, suitable for representation and evaluation, see e.g. [9–12]. A technique that is based on such segmentation has been proposed in [13] to extract a discontinuity feature from sleep EEG. Within this technique detected pseudo-stationary intervals were used for estimating the average amplitudes which then form an Amplitude Vector (AV). Statistics derived from distributions of AV were found correlated with the EEG maturation of newborns between 25 and 35 weeks post-conception. However, these statistics varied largely between patients.

An alternative approach, proposed in our previous work [14], aimed at estimating the EEG discontinuity as a *rate* of pseudo-stationary segments. This technique detected EEG intervals within which the statistics of spectra powers were changed insignificantly. The calculated statistics were compared in adjacent intervals of EEG. The new feature was correlated with newborn age and shown to be capable of increasing the accuracy of classification between pre-term and full-term newborns, respectively.

The above work was undertaken within a methodology of Bayesian Model Averaging (BMA) aimed at estimating the full predictive posterior probability distribution that is required for accurate estimation of uncertainty intervals, see e.g. [15]. The use Decision Tree (DT) models within BMA provides selection of predictors that are important for classification, see e.g. [16, 17]. DT models provide experts with new insights into data and interpretation of decision making. A single DT model can be selected for interpretation purposes as shown in our work [18].

The Bayesian averaging over DT models is practically implemented with the Markov Chain Monte Carlo (MCMC) method aimed at exploring a posterior density of model parameters by making random walk proposals, see e.g. [17, 19]. The MCMC methods have been recently applied for modelling and simulation problems in biomedicine, see e.g. [20, 21] including Bayesian analysis of EEG [22].

In this paper we explore the EEG discontinuity feature used along with the spectral power characteristics within the Bayesian classification of newborn development in 10 age groups between 36 and 45 weeks. The proposed technique is compared with the conventional discontinuity techniques [8, 13] based on the threshold and adaptive segmentations in terms of correlation with newborn age, classification accuracy and uncertainty. We also compare our technique with the adaptive segmentation [10] that is based on autoregressive modelling.

The rest of the paper is structured as follows. We discuss the techniques of extracting EEG discontinuity features and describe a new approach. Then we describe our methodology and experiments and explore the correlation of the conventional and new discontinuity features with newborn brain maturity. We show that the new features are more strongly correlated with brain maturation. We also compare the new features for Bayesian classification of EEG obtained in 10 age groups in terms of age classification accuracy. Finally we show that the new features provide more accurate assessments of EEG maturation. The S1 Appendix provides details of the Bayesian method.

## Extraction of EEG features

In this section we analyse the feature extraction methods based on adaptive segmentation, that were developed for detecting boundaries of pseudo-stationary EEG intervals. Finally we describe our approach to feature extraction.

### Adaptive segmentation for extracting EEG features

In [9], boundaries of quasi-stationary intervals in a signal *x*(*n*) are detected by using an autoregressive (AR) model given with parameters *ω* for modelling homogeneous parts of the signal *x*. It has been shown that changes in parameters *ω* that are adjusted to different intervals define boundaries of interest. A given AR model generates the outcome $\hat{y}\left(n,\omega \right)$ as follows

$$\begin{array}{c} \hat{y}\left(n,\omega \right)=\sum_{k=1}^{p}\omega \left(k\right)x\left(n-k\right)-x\left(n\right), \end{array}$$ (1)

where *ω*(*k*) are the coefficients and *p* is the order of AR model.

Signal *x*(*n*) is modelled in the reference and test windows. The modelling errors $e\left(n\right)=\hat{y}\left(n\right)-x\left(n\right)$ are hypothesised to be a white noise process on a homogeneous part of *x*(*n*).

Based on the above approach, the errors *e*(*n*) calculated in a window are hypothesised to be distributed as white noise. Such a hypothesis is tested with *Z*-statistic as describe in [10]. The overall *Z*-statistic is combined over the reference, *R*, and test, *T*, windows as follows

$$\begin{array}{c} Z=Z(I|J)+Z(J|I), \end{array}$$ (2)

where *Z*(*I*|*J*) are the statistics of cross-validation errors calculated for windows *I* ∈ {*T*, *R*} and *J* ≠ *I*.

The statistics *Z*(*I*|*J*) are defined as follows

$$\begin{array}{c} Z\left(I|J\right)=\left|\frac{1}{2N_{I}}\sum_{n=1}^{N_{I}}\left(\frac{e_{I}{\left(n\right)}^{2}}{\sigma_{J}^{2}}-1\right)\right|, \end{array}$$ (3)

where *N*_*I*_ is the size of window *I*, *e*_*I*_(*n*)^2^ is the residual error, and $\sigma_{J}^{2}$ is the variance of estimated noise in the window *J*.

The cross-validation error *e*_*I*_(*n*)^2^ in Eq 3 is calculated for an AR model with coefficients *ω*_*J*_ fitted to the window *J*, so that *e*_*I*_(*n*)^2^ is

$$\begin{array}{c} e_{I}{\left(n\right)}^{2}={\left({\hat{y}}_{I}\left(n,\omega_{J}\right)-x_{I}\left(n\right)\right)}^{2}. \end{array}$$ (4)

In the above Eq 3 the variance $\sigma_{J}^{2}$ is calculated for an AR model with parameters *ω*_*J*_, so that $\sigma_{J}^{2}$:

$$\begin{array}{c} \sigma_{J}^{2}=\frac{1}{N_{J}-p}\sum_{n=p+1}^{N_{J}}{\left({\hat{y}}_{J}\left(n,\omega_{J}\right)-x_{J}\left(n\right)\right)}^{2}. \end{array}$$ (5)

The *Z*-statistic defined in Eq 2 is calculated for each pair of the reference and test windows and then compared with a critical value, *Z*_*cr*_. For EEG signals, *Z*_*cr*_ is found empirically.

A similar approach was adopted in [13, 23] for extracting EEG features. In particular, the adaptive segmentation is used for generating an amplitude vector, proposed in [13], in order to extract the discontinuity feature.

The above techniques were implemented for our experiments as Matlab scripts included in the Supporting Information.

### Extraction of discontinuity feature from amplitude vector

According to [13], the discontinuity feature is extracted from an amplitude vector (AV) generated from a segmented EEG as follows. First, the mean *μ*_*i*_ of absolute amplitudes is computed for each pseudo-stationary segment, *i* = 1, 2, …, including *L*_*i*_ samples. The value *μ*_*i*_ is then repeated *L*_*i*_ times. For examples, given *L*_*i*_ = 600, the value *μ*_*i*_ is repeated 600 times. At the second step, a distribution of the generated AV is estimated and then approximated with a log-normal distribution. Finally, the location *μ* and scale *σ* of this distribution represent the features of interest.

**Fig 1:**
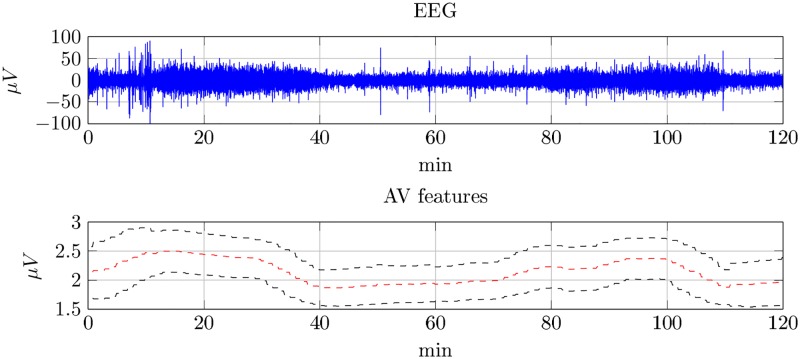
Amplitude variability over sleep stages. a) A 120-min sleep EEG recorded from a newborn at age of 44 weeks, b) *μ* (Red) and *σ* (Black) are the parameters of the distribution of AV extracted from EEG.

Fig 1 illustrates how discontinuity features are changed during sleep of a newborn at age of 44 weeks. Here the feature is represented by a location *μ* and a scale *σ* computed in a 10-min window sliding with a 1-min step over a 120-min recording. The intervals between 10 and 40 min as well as between 80 and 110 min, identified as the quiet sleep phase, are with a high discontinuity value. In contrast, the active phase, that is between 40 and 80 min as well as between 110 and 120 min, is with a low discontinuity value.

### Proposed feature extraction technique

In [24], a technique proposed for estimating the stationarity of EEG signals has employed the spectral density function calculated in two separate intervals. The spectral densities estimated in these intervals are then compared within a 2-sample Kolmogorov-Smirnov (KS) test. This technique was used to estimate the stationarity of intervals when their lengths varied between 1 and 64 sec.

A similar approach, based on a statistical test, is adopted in our technique in order to extract the discontinuity feature. The proposed technique based on the Spectral Power Statistics (SPS) is described below.

Algorithm 1 describes the main steps of the proposed segmentation technique. The reference *W*_1_ and test *W*_2_ windows are sliding along a signal *X*. The length of both windows is given by *L*. For each position of the windows *W*_1_ and *W*_2_, Fast Fourier Transform (FFT) computes the spectral powers *S*_1_ and *S*_2_ within a given frequency band *S*. These powers are used for testing a hypothesis that EEG signals in the reference and test windows are from the same quasi-stationary process within a given critical level *d*_0_.

For given signal *X*, length *L*, band *S*, and value *d*_0_, the Algorithm 1 finds boundaries of interest and returns their indexes as a vector *T*. At lines 9 and 10 the indexes of reference *W*_1_ and test *W*_2_ windows are assigned. At the next lines 11 and 12, the spectral powers *S*_1_ and *S*_2_ are calculated for windows *W*_1_ and *W*_2_, respectively. If a distance *d* of the KS test exceeds the critical value *d*_0_, the EEG signals in windows *W*_1_ and *W*_2_ have different characteristics, and the line 15 assigns a boundary of the pseudo-stationary segment to the output vector *T*.

In our experiments we achieved the best segmentation with the following parameters: length *L* = 200 samples, that is a 2-sec duration given a sampling frequency *F* = 100 Hz, a value *d*_0_ = 0.15, and a frequency band *S* = (0, 13.5) Hz. Given *F* = 100 Hz, the band *S* is represented by 28 spectral lines that is a sufficient sample size for the statistical KS test.

**Algorithm 1** Adaptive segmentation using Spectral Power Statistics

1: **Inputs**: *X*, *L*, *S*, *d*_0_

2: **Initialise**:

3: *i*_1_ ← 1                 ▷ Reference window index

4: *i*_2_ ← *i*_1_ + *L*                  ▷ Test window index

5: *L*_1_ ← *L* − 1

6: *K* ← *floor*(*length*(*X*)/*L*) − 1          ▷ Number of segments

7: *T*[1, *K*] ← 0                 ▷ Segmentation vector

8: **for** *k* ← 1, *K* **do**

9:   *W*_1_ ← [*i*_1_, *i*_1_ + *L*_1_]              ▷ Reference window

10:  *W*_2_ ← [*i*_2_, *i*_2_ + *L*_1_]                ▷ Test window

11:  *S*_1_ ← *Sum*(FFT(*X*(*W*_1_)), *S*)          ▷ Spectral powers

12:  *S*_2_ ← *Sum*(FFT(*X*(*W*_2_)), *S*)

13:  *d* ← StatTest(*S*_1_, *S*_2_)              ▷ Statistical test

14:  **if** *d* > *d*_0_ **then**

15:   *T*[*k*] ← *i*_2_             ▷ A new segment boundary

16:  **end if**

17:  *i*_1_ ← *i*_1_ + *L*                 ▷ Moving windows

18:  *i*_2_ ← *i*_2_ + *L*

19: **end for**

20: **return** *T*

### New discontinuity feature

Having recorded the locations of segment boundaries in the vector *T*, we can consider a rate of pseudo-stationary intervals as a discontinuity feature and introduce a segmentation rate, *sr*, as follows:

$$\begin{array}{c} sr=K{\left[\frac{∥X∥}{L}\right]}^{-1}, \end{array}$$ (6)

where *K* is the number of pseudo-stationary segments detected in a signal *X* and stored in *T*, $\left[\frac{∥X∥}{L}\right]$ is the maximal number of segments that can be detected in signal *X* by using a window of length *L*, and ‖*X*‖ is the length of *X*.

**Fig 2:**
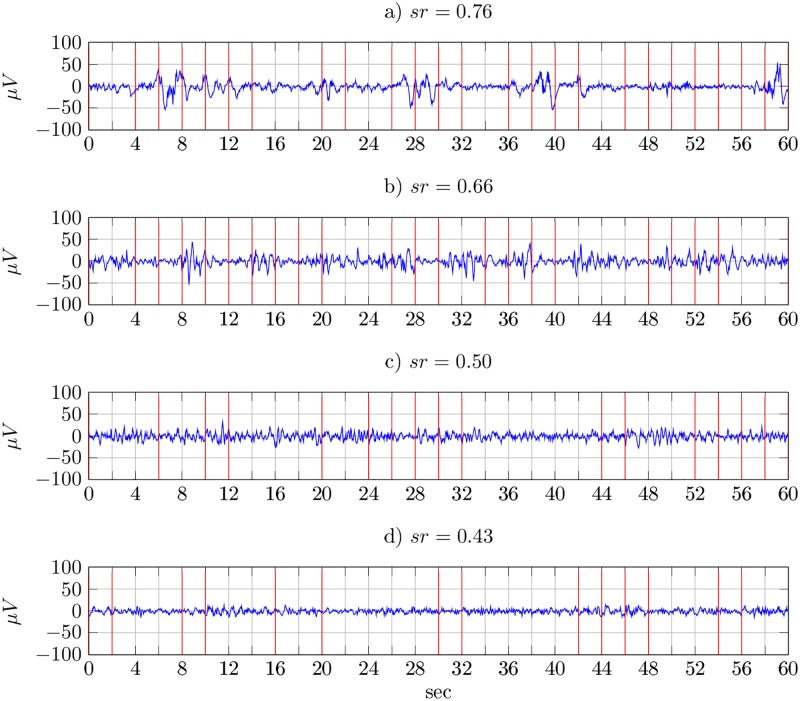
Segmentation results. Segment rates, *sr*, for different EEG patterns: a) discontinuous pattern at 36 weeks, b) semi-discontinuous pattern at 38 weeks, c) and d) continuous patterns at 41 weeks.

According to Eq 6, the larger the *sr* value, the larger is the number *K* of segments and, therefore, higher is the discontinuity of sleep EEG. Fig 2 shows the results of the proposed segmentation technique, where the boundaries of pseudo-stationary segments are labelled by the vertical bars in Red. The *sr* is higher for the EEG recorded at 36 and 38 weeks, shown on plots a) and b). For the EEG recorded at 41 weeks shown on plots (c) and (d), the variations in EEG activity are smaller and so segment rate *sr* is decreased.

## Experiments with EEG data

In this section we present results of our experiments on the EEG data recorded during sleep hours from newborns in 10 age groups. We explore the correlation of the proposed discontinuity feature with the newborn ages. Finally we compare the proposed and existing discontinuity features in terms of classification and uncertainty estimation accuracy.

### Description of EEG data

In our experiments we used 1,110 EEG recorded from newborns in 10 age groups from 36 to 45 weeks, with approximately 100 recordings in a group. The data were recorded during the project on automated EEG assessment of newborn brain development, see e.g. [25, 26], conducted at the University of Jena, Germany.

The recordings were made with the C3-T3 and C4-T4 electrodes with a sampling rate *F* = 100 Hz. The electrodes were positioned according to the standard 10–20 electrode system. Raw EEG were filtered to remove slow drifts with frequencies below 0.1 Hz and noise along with high-frequency interference above 30 Hz. The EEG segments with amplitudes that exceeded a threshold found as ±1.5 standard deviation of amplitudes in a 2-min sliding window were removed as artefacts. Segments with the spectral power below 10% of the average power were also removed as “lost” signal. The average rate of artefacts was around 20%.

The EEG were analysed in the standard frequency bands that are typically used for analysis of sleep EEG. Table 1 shows the six standard bands and their frequency ranges.

**Table 1:** EEG frequency bands.

#	Band	Range, Hz
1	Subdelta	0–1.5
2	Delta	1.5–3.5
3	Theta	3.5–7.5
4	Alpha	7.5–13.5
5	Beta1	13.5–19.5
6	Beta2	19.5–25

Standard frequency bands for analysis of sleep EEG.

### Methodology of experiments

The above data were used in our experiments for comparison of the proposed and existing techniques described in the previous section. The features extracted from segmented EEG were compared, first, in terms of correlation with newborn ages and, second, in terms of accuracy of age classification and uncertainty estimation.

#### Correlation with brain development

The AR model based segmentation technique described in the above section was run with the reference and test windows being set with 2-sec duration and a 2-sec moving step similar to the SPS technique. In our experiments we applied *Z*_*cr*_ ∈ (4.0, 9.0) and obtained almost the same correlation with ages, *ρ* ≈ 0.60.

A threshold (TR) segmentation technique, proposed in [8], calculates a difference, *d*_*k*_:

$$d_{k}=\max_{1\leq n\leq N}\left(x_{n}\right)-\min_{1\leq n\leq N}\left(x_{n}\right),k=1,\ldots ,K,$$

where *N* is the number of EEG samples in the *k*th interval of 2-sec duration, and *K* is the number of the intervals in EEG.

Differences *d*_*k*_ are calculated for all *K* intervals and then compared with a threshold *d*_0_ ∈ {25, 50}*μV*:

$$\begin{array}{c} d_{k}-d_{0}\left\{\begin{array}{cc} >0, & T_{k}=1,\;\text{continuity},\\ \leq 0, & T_{k}=0,\;\text{discontinuity.} \end{array}\right. \end{array}$$ (7)

Then, finally, a ratio of the continuous intervals, $\sum_{k=1}^{K}\left(T_{k}|T_{k}=1\right)/K$, is considered as a discontinuity feature along with the segmentation vector *T*. If *T*_*k*_ + 1 ≠ *T*_*k*_, then a boundary is assigned between segments *k* and *k* + 1, otherwise the segments are considered to be similar.

The proposed SPS technique was run with the reference and test windows of 2-sec duration, each including *L* samples. The windows were set to be moving with a 2-sec step. The frequency band *S*, in the Algorithm 1 was set in the range (0, 13.5) Hz, that includes the standard bands Subdelta to Alpha, shown in Table 1. The critical value *d*_0_ for the KS test was given 0.15. This value enabled the algorithm to assign a segment boundary if the spectral powers *S*_1_ and *S*_2_, that are considered to be sampled from the same stationary process, are different with a *p*-value, *p* < 0.9.

#### Classification of EEG maturity

In experiments we used Bayesian method to compare the assessment accuracy that can be obtained with the proposed and conventional EEG features. Bayesian methods are known for accurate estimation of predictive posterior probabilities, *P*_*ij*_, for each input *i* and each class *j*. This enables practitioners to reliably estimate the uncertainty intervals for each patient. The S1 Appendix provides details of the Bayesian method.

The above predictive posterior probabilities are calculated in our experiments with different feature extraction techniques in order to estimate and compare uncertainties of age classification. Following [27], the uncertainty is estimated in terms of Entropy, *E*, as follows

$$\begin{array}{c} E=-\sum_{i=1}^{T}\sum_{j=1}^{C}P_{ij}log_{2}\left(P_{ij}\right), \end{array}$$ (8)

where *T* is the size of test data that are used for analysing the predictive accuracy, and *C* = 10 is the number of age groups.

The EEG were recorded from newborns in 10 age groups between 36 and 45 weeks. Each group was represented by approximately 100 EEG recordings. Because of physiological variability, sleep EEG are difficult to distinguish, and assessments are made within ±1 week of the post-conceptual age. The accuracy of such assessment provided by EEG experts, known from [4], is 65.0%, that is the baseline for our comparison.

### Experimental results

Table 2 shows performances of the SPS, AR and TR segmentation techniques in terms of correlation observed between the extracted features and post-conceptional ages. The correlation was estimated with Spearaman’s rank correlation coefficient, *ρ*. The columns *AV*_*μ*_ and *AV*_*σ*_ show correlations *ρ* obtained by the AV technique when the EEG were segmented by the SPS, AR and TR techniques. The results achieved with the TR techniques were obtained for 25*μV* and 50*μV* threshold and denoted TR(25) and TR(50), respectively.

**Table 2:** Correlation of EEG features.

Segmentation technique	Correlation, *ρ*		
	*AV μ*	*AV σ*	*sr*
SPS	0.384	0.113	−**0.734**
AR	0.385	0.093	-0.598
TR(25)	0.378	0.099	-0.293
TR(50)	0.384	0.223	0.245

Correlation, *ρ*, of the extracted EEG features with post-conceptional age.

The columns *AV*_*μ*_ and *AV*_*σ*_ in Table 2 show the correlation obtained with the location *μ* and scale *σ*, that were estimated by the AV technique, respectively. The last column, *sr*, shows the results obtained with the feature *sr*, defined by Eq 6, for all the segmentation techniques.

In Table 2 we see that the proposed SPS technique has extracted the new feature with the strongest correlation, *ρ* = −0.734. The second result, *ρ* = −0.598, was obtained with the AR segmentation technique. The TR(50) techniques, applied for segmentation with a 50*μV* threshold, provided the weakest correlation, *ρ* = 0.245. At the same time, the ratios of segments with EEG activity exceeding a given threshold are correlated with age, delivering *ρ* = 0.344 and *ρ* = 0.302 for 25*μV* and 50*μV* thresholds, respectively. All results were statistically significant with *p*-value, *p* < 0.01.

**Fig 3:**
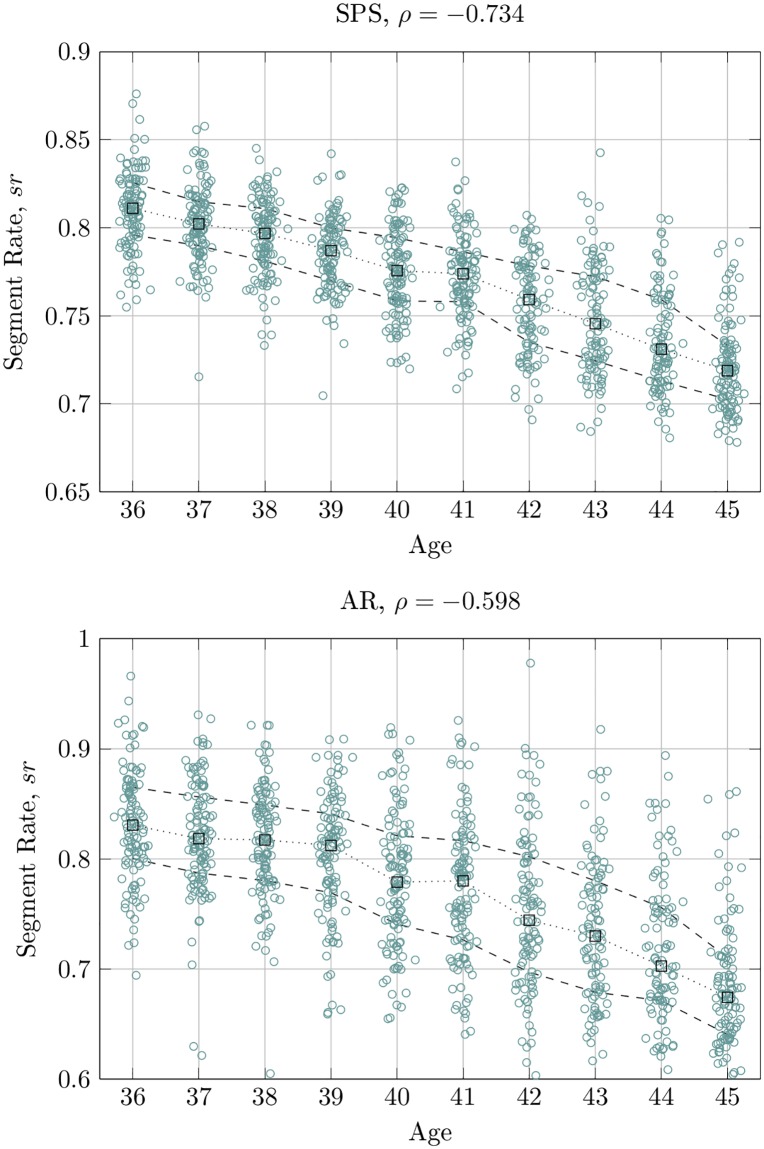
Correlation of the *sr* features extracted by the SPS and AR techniques. The circles represent *sr* values calculated for an EEG recording. The squares represent the median, and the dashed lines denote the 25^*th*^ and 75^*th*^ percentiles.

Observing the correlations *ρ* in the column *sr*, we see that the rates of segments are decreased with post-conceptional age for the SPS, AR, and TR(25) techniques, and *ρ* < 0. For the TR(50) segmentation with a 50*μV* threshold the tendency is opposite and *ρ* > 0. This can be explained by a higher EEG activity allowed in segments that reflects the fact of increasing EEG activity with newborn age. Fig 3 shows the correlation between newborn age and *sr* obtained with the proposed SPS and AR techniques.

Table 3 shows the performance, *P*, and entropy *E*, calculated by Eq 8, for the Bayesian classification using EEG features extracted with the SPS, AR, and AV techniques.

**Table 3:** Performances and entropies of Bayesian classification.

Technique	*P*, %	*E*
SPS	69.2 ± 0.8	199.3 ± 10.5
AR	65.3 ± 0.8	205.1 ± 11.7
AV	63.5 ± 0.7	218.6 ± 8.4

Performances of Bayesian classifications using EEG features extracted with the SPS, AR, and AV techniques.

The average performance and 2*σ* intervals were calculated within the 10-fold cross validation. We observe that the average performance of the SPS technique is 69.2% that is higher than that provided by the AR techniques. Moreover, the new feature provides a smaller classification uncertainty, giving an entropy *E* = 199.3.

## Conclusion

EEG discontinuity is known in the literature as an important feature for evaluating brain development of newborns in weeks between 28 and 42 weeks of post-conceptional age. The conventional approach is based on discontinuity features that can be extracted from segmented EEG.

In our research we found that the discontinuity features, extracted within the existing approaches, become weakly correlated with brain maturity at 36 and 45 weeks, that affects the assessment accuracy. This observation inspires us to assume that more accurate results can be achieved with a new discontinuity feature estimated as a rate of pseudo-stationary intervals which can be detected by a new adaptive segmentation technique. We hypothesised that such a feature will be more strongly correlated with brain maturation. Our assumption was based on the observation that during brain development the continuous EEG patterns become longer, while the discontinuous patterns become shorter, and this increases a correlation between the proposed feature and age-related changes.

The proposed and conventional features were compared on the EEG data recorded from newborns in 10 age groups from 36 to 45 weeks. In our experiments we found that the new features provide a stronger correlation with ages. The new EEG features were explored within the Bayesian assessment of brain development. The new features have improved the assessment accuracy achieving 69.2%, whilst the accuracy of the baseline expert evaluation known from the literature is 65.0%. The existing feature extraction techniques were incapable of exceeding the baseline accuracy.

It is also important to note that predictive distributions generated by the Bayesian method are used to provide an accurate approximation of uncertainty intervals within which a prediction is distributed. This becomes critically important when technologies assist practitioners to avoid fatal errors.

## Supporting information

S1 Appendix Bayesian method. (PDF)
