# Supplementary material for: Extraction of features from sleep EEG for Bayesian assessment of brain development
Source: PLoS One. 2017 Mar 21;12(3):e0174027. doi: 10.1371/journal.pone.0174027 (PMC5360314; doi:10.1371/journal.pone.0174027)
Supplement: S1 Appendix — (PDF) [file pone.0174027.s001.pdf]

S1 Appendix. Bayesian Method.

We are interested in the predictive distribution analytically written as an integral over parameters of a DT model

$$p(y|x, \mathbf{D}) = \int p(y|x, \Theta, \mathbf{D}) p(\Theta|\mathbf{D}) \mathrm{d}\Theta, \tag{1}$$

where y is the predicted class $y \in \{1, C\}$, $x = (x_1, \dots, x_m)$ is the m-dimensional input vector, and $\mathbf{D} = (x^{(i)}, y_i)_{i=1}^{n}$, is the set of n labelled (or training) data points.

This integral is analytically tractable only in simple cases. The posterior density of Θ conditioned on $\mathbf{D}$, $p(\Theta|\mathbf{D})$, can be also evaluated in very simple cases. Using MCMC integration, we can draw $\Theta^{(1)} \dots, \Theta^{(i)}$ from the posterior distribution of $\Theta|\mathbf{D}$, and then write

$$\begin{aligned} p(y|x, \mathbf{D}) &\approx \sum_{i=1}^{N} p(y|x, \Theta^{(i)}, \mathbf{D}) p(\Theta^{(i)}|\mathbf{D}) \\ &= \frac{1}{N} \sum_{i=1}^{N} p(y|x, \Theta^{(i)}, \mathbf{D}), \end{aligned} \tag{2}$$

where N is the number of samples that are taken from the posterior distribution.

The above integration is achieved when we can generate random samples from $\Theta|\mathbf{D}$ by running a Markov Chain having a stationary distribution. Starting from an arbitrary state, the Markov chain has to converge to a stationary one during the burn-in phase. Then during the post burn-in phase samples from the Markov Chain are collected to calculate the desired posterior density.

MCMC integration over models of variable dimension of Θ is achieved by using the Reversible Jump extension described in [1].

In order to use DT models for Bayesian averaging, first we need to determine the probability φ_{ij} with which the ith terminal node, $i = (1, k)$, assigns an input x to the jth class, where k is the number of terminal nodes in the DT.

Then we can define DT parameters as a vector $\Theta = (s_i^{pos}, s_i^{var}, s_i^{rule}), i = 1, \dots, k-1$. Here, s_i^{pos} denotes the ith node position in the DT, s_i^{var} defines the variable used at the node for splitting, and s_i^{rule} specifies the threshold value of this variable.

The above notations allow us to define the following priors on DT models. Given a number of the minimal data points, p_{min}, that are allowed in splitting nodes, first we expect that a maximal number of splitting nodes $s_{max} = n/p_{min}$, where n is the number of data points in the D. Then we can assign node i to be drawn from a uniform distribution, $s_i^{pos} \sim U(1, s_{max})$.

Similarly, we can assign any of the m variables drawn from a uniform discrete distribution, $s_i^{var} \sim U(1, m)$. Finally, we can assign the threshold value to be drawn from a uniform discrete distribution, $s_i^{rule} \sim U(z^{(1)}, \dots, z^{(K)})$, where K is the number of possible splitting rules for predictor $z = s_i^{var}$.

The above priors allow us to generate DT models of different configurations which split the data in many ways. Ideally, each DT with the same number of terminal nodes can be explored equally likely [2]. For this case the prior for a

DT is written as

$$p(\Theta) = \left\{ \prod_{i=1}^{k-1} p(s_i^{rule}|s_i^{var}) p(s_i^{var}) \right\} p(\{s_i^{pos}\}_1^{k-1}) \tag{3}$$

In some cases, there is the knowledge of the favoured DT configuration. For example, the prior probability of further splits of the terminal nodes can be defined dependent on how many splits have already been made above them, see e.g. [3]. The use of such information allows MH algorithm explores the favoured DT models in more details.

In practice, DT models are typically grown to a large size. The MCMC integration over large DT models of variable dimensionality is performed with the following types of moves, see e.g. [1, 3, 2]:

1. *Birth.* Randomly split the data points falling in one of the terminal nodes by a new splitting node with the variable and rule drawn from the given priors.
2. *Death.* Randomly pick a splitting node with two terminal nodes and assign it to be a terminal one with the united data points.
3. *Change-split.* Randomly pick a splitting node and assign it a new splitting variable and rule drawn from the given priors.
4. *Change-rule.* Randomly pick a splitting node and assign it a new rule drawn from a given prior.

The first two moves, birth and death, are reversible and change the dimensionality of the vector Θ. The remaining moves provide jumps within the current dimensionality of Θ. The change-split move is included to make "large" jumps which potentially increase the chance to sample from maximal posterior, whilst the change-rule move makes "local" jumps to allow the neighbourhood areas of interest to be explored in detail.

For the birth moves, the proposal ratio R is written as

$$R = \frac{q(\Theta|\Theta')p(\Theta')}{q(\Theta'|\Theta)p(\Theta)}, \tag{4}$$

where $q(\Theta'|\Theta)$ is the proposal distributions of moving from the proposed parameters Θ' to the current Θ, $q(\Theta'|\Theta)$ is the probability of the reverse move, Θ' and Θ are $(k+1)$ and k-dimensional vectors of DT parameters, respectively, and $p(\Theta)$, $p(\Theta')$ are the probabilities of the DT models with parameters Θ:

$$p(\Theta) = \left(\prod_{i=1}^{k-1} \frac{1}{N(s_i^{var})} \frac{1}{m} \right) \frac{k}{S_k} \frac{1}{K}, \tag{5}$$

where $N(s_i^{var})$ is the number of values of s_i^{var} which can be assigned as a new splitting rule, S_k is the number of ways of constructing a DT with k terminal nodes, and K is the maximal number of terminal nodes, $K = s_{max} + 1$.

For binary DT models, the number S_k is defined by the Catalan number

$$S_k = \frac{1}{k+1} \binom{2k}{k}, \tag{6}$$

and we can see that for $k \geq 25$ the number of possible DT models becomes very large, $S_k \geq 4.8^{12}$.

The above proposal distributions are defined as follows:

$$p(\Theta|\Theta') = \frac{d_{k+1}}{D_{Q1}}, \tag{7}$$

$$p(\Theta' p|\Theta) = \frac{b_k}{k} \frac{1}{N(s_i^{var})} \frac{1}{m}, \tag{8}$$

where $D_{Q1} = D_Q + 1$ is the number of splitting nodes whose branches are both terminal nodes.

Finally, the ratio R_b for a birth move is written as

$$R_b = \frac{d_{k+1}}{b_k} \frac{k}{D_{Q1}} \frac{S_k}{S_{k+1}}. \tag{9}$$

The number D_{Q1} is dependent on the DT structure and so expected that $D_{Q1} < k, k = 1, \ldots, K$.

In particular, when $k = 2, \ldots, K{-}1$, we can assume $d_{k+1} = b_k$. Then $k \to K$, DT models are growing, and $S_{k+1} > S_k$, so that a proposal ration R becomes between 0 and 1.

Alternatively, for the death moves the ratio R_d is

$$R_d = \frac{b_k}{d_{k-1}} \frac{D_Q}{(k-1)} \frac{S_k}{S_{k-1}}. \tag{10}$$

Because DT models are hierarchical structures, changes to the nodes located close to the root can cause drastic changes to the distribution of data points at the lower levels. For this reason there is a very small chance to accept the change in a node near the DT root. This means that MH algorithm will tend to explore the DT models in which the nodes chosen to be changed are far from the root node. These nodes typically contain small numbers of data points, and consequently, the value of likelihood is not changed much, and such moves are mostly accepted.

The negative impact of this is that MH algorithm cannot explore the full posterior distribution of DT models, see e.g. [2]. The DT models grow very quickly during the first burn-in samples because an increase in the log likelihood value for the birth moves is much larger than that for the other moves, and almost every birth move is accepted.

Once a DT has grown, the changes in nodes near to the DT root have very small acceptance probability, and MH algorithm tends to get stuck in particular DT structures.

A new prior suggested in [4] was capable of controlling the overgrowth of DT models by attempting to reduce the probability of proposing excessive birth moves for a given split. Using this prior, MH algorithm assigns a proposal from a uniform distribution whose parameters are estimated on data points that fall into the split. The use of the new prior has improved the results of Bayesian averaging over DT models on the benchmark data sets as well as on the real-world problems.

References

[1] Green PJ. Reversible Jump Markov Chain Monte Carlo and Bayesian model determination. Biometrika. 1995;82:711–732.

[2] Denison D, Holmes C, Mallick B, Smith A. Bayesian Methods for Nonlinear Classification and Regression. Wiley; 2002.

[3] Chipman H, George E, McCullock R. Bayesian CART model search. Journal of American Statistics. 1998;93:935–960.

[4] Schetinin V, Fieldsend JE, Partridge D, Krzanowski WJ, Everson RM, Bailey TC. The Bayesian Decision Tree Technique with a Sweeping Strategy. In: The International Conference on Advances in Intelligent Systems. IEEE Computer Society; 2004.
